# Pediatric 3D MRCP imaging: strategies for enhancing exam quality

**DOI:** 10.1007/s00261-025-05063-y

**Published:** 2025-06-27

**Authors:** Mohammad Jalloul, Sudha A. Anupindi, Shyam S. B. Venkatakrishna, Abhay S. Srinivasan, Michael R. Acord, Jorge Delgado, Levy C. Onyango, Youck Jen Siu Navarro, Janet R. Reid, Rebecca Dennis, Valerie A. Rigby, Summer L. Kaplan, Suraj D. Serai

**Affiliations:** Children’s Hospital of Philadelphia, Philadelphia, USA

## Abstract

MRCP is essential for noninvasive evaluation of the biliary and pancreatic ductal systems in children but can suffer from suboptimal image quality due to inconsistent protocols and technical factors. We performed a quality improvement project focused on enhancing the image quality of 3D MRCP at a tertiary children’s hospital. The project identified key contributors to poor image quality, including the inconsistent use of respiratory-triggered techniques and the absence of standardized protocols across multiple MRI units. Interventions were implemented, including updating protocols, technologist education, and improving communication between radiologists and technologists. We set a goal of achieving 90% of MRCP exams with acceptable image quality by September 2024. Our efforts increased the success rate from 68 to 77%. Although the target was not fully reached by the end of the set project timeline, the effort highlights the importance of multidisciplinary collaboration, continuous education, and ongoing auditing in driving improvements in imaging quality.

## Introduction

Magnetic resonance cholangiopancreatography (MRCP) is a noninvasive imaging technique that visualizes the biliary and pancreatic ducts. It is often used to diagnose several conditions in children, including primary sclerosing cholangitis, congenital abnormalities, choledocholithiasis, and both acute recurrent and chronic pancreatitis [1, 2].

The development of 3D T2-weighted fast-spin-echo (FSE) techniques acquired with isotropic or near-isotropic resolution has afforded MRCP high clinical utility such as in delineating the pancreaticobiliary junction in infants and young children [3]. SPACE (sampling perfection with application optimized contrasts using different flip angle evolutions), enables acquisition of high-spatial-resolution 3D MRCP images, producing high-quality imaging of the biliary system on 1.5T and 3T scanners [4, 5]. However, several factors influence the quality of MRCP images, including patient-specific characteristics and technical aspects related to the MRI system and acquisition parameters. Patient-specific parameters, such as body habitus, physical movement, and respiratory motion, can significantly impact image quality. Technical parameters, including artifacts, high signal-to-noise ratio, and pulse sequence settings also play a crucial role [6]. Understanding and modifying these factors beforehand could help prevent nondiagnostic MRCP exams.

In this Quality Improvement (QI) project, our team aimed to enhance MRCP image quality by identifying and addressing key factors that influence image clarity: minimal artifacts, adequate background suppression on the 3D SPACE sequence, and clearly visible ducts (common bile duct, hepatic ducts, and pancreatic duct). By engaging radiologists, technologists, MR physicists, and other stakeholders, our goal was to increase the percentage of diagnostic-quality MRCP images from a baseline of 68% in May 2023 to 90% by September 2024.

## Methods

### MRCP protocol

All patients underwent an MRI including a 3D FSE MRCP acquisition on either a 1.5-T (Avanto, Siemens) or 3-T (Vida, Siemens; or Prisma, Siemens) MRI scanner at *(masked)*. None of the patients had received oral preparations for the MRI examination. Coronal 3D FSE MRCP thin-section source images, along with scanner-generated 3D maximum-intensity-projection (MIP) thick-slab images, were archived on the PACS system. The 3D FSE MRCP sequence details are presented in Table 1.

**Table 1:** Parameters for 3D fast Spin-Echo MRCP acquisition

Parameter	Value
Pulse sequence	3D FSE (SPACE)
Slice thickness (mm)	1
Matrix	320 × 225
Echo time (TE) (ms)	702
Repetition time (TR) (ms)	2500
Bandwidth (Hz/pixel)	501
No. of signals averaged (NEX/ NSA)	1.6
Acceleration factor	2
Flip angle (degrees)	140
Fat saturation	Yes
Scan time	Approximately 4.5 min

Respiratory movement reduces MRCP image quality by increasing noise, producing “ghost” artifacts, and by decreasing edge sharpness in moving structures. Multiple techniques exist within the MRCP protocols to mitigate these effects including breath-hold and respiratory triggering techniques [7].

**Fig. 1:**
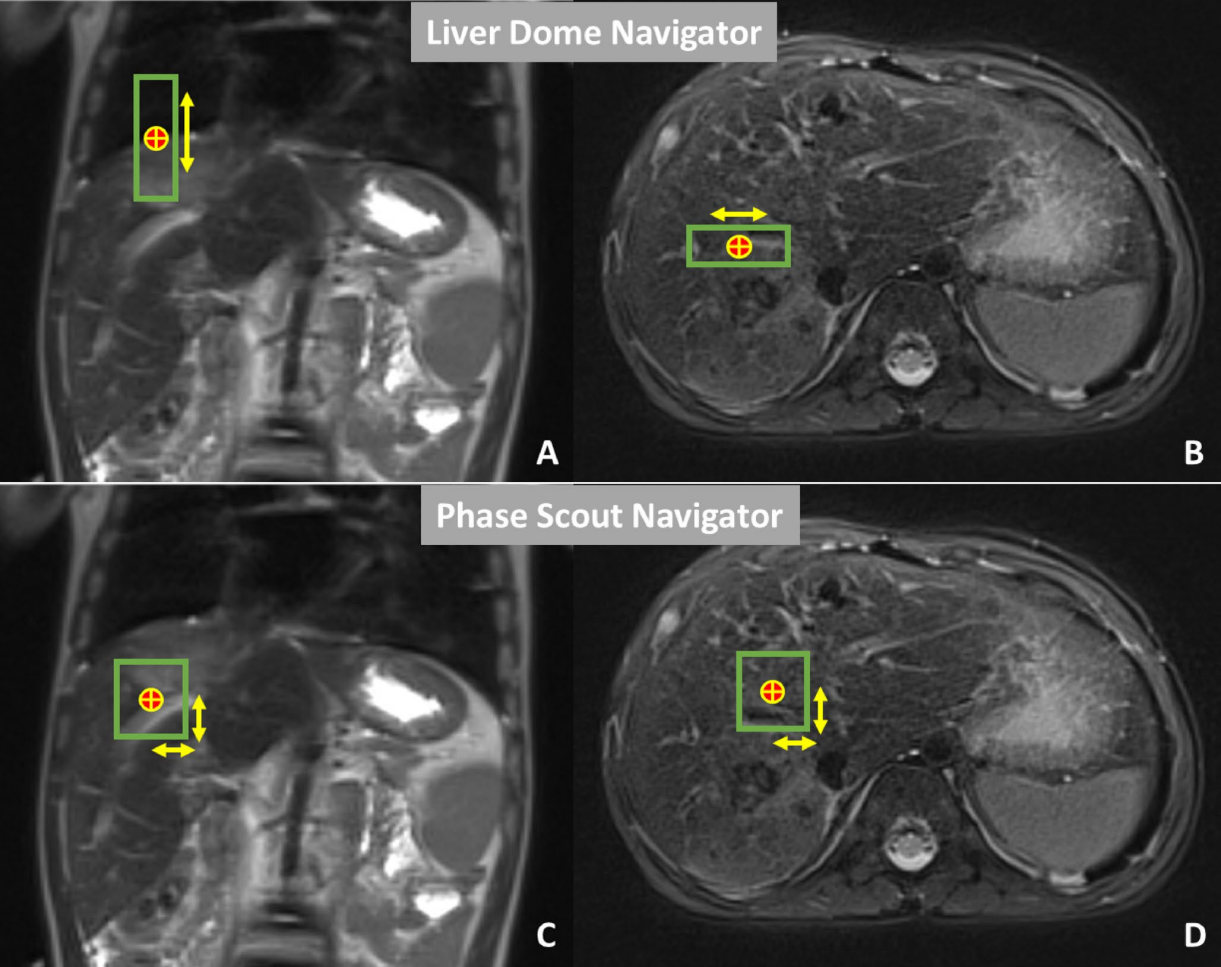
Motion Navigator techniques. The red and yellow circle reflects the region of interest within the liver indicating the navigator pulse sequence. The green borders represent the areas of motion. “**A**” & “**B**” reflect “Liver Dome” navigator technique settings and “**C**” & “**D**” represent phase scout navigator technique settings

“Respiratory Triggering” initiates MR image acquisition at a fixed point in the respiratory cycle, improving edge sharpness and reducing ghost artifacts [8]. The “Motion Navigator” technique integrates a navigator pulse sequence into the imaging sequence to monitor a region of interest (ROI) in a relatively stable area, such as the diaphragm or liver. When motion is detected, the system adjusts imaging in real time. This technique utilizes 2 different types of navigators. The “Liver Dome” navigator places the ROI over the liver dome, spanning both the liver and chest, to monitor breathing motion, triggering imaging only within a defined motion range. The “Phase Scout” navigator captures a low-resolution image to determine the patient’s respiratory phase to align the image sequences to a consistent point in the respiratory cycle, ensuring imaging occurs during the most stable respiratory phase [9] (Fig. 1).

### Ethical considerations

This work was performed as quality improvement and therefore was not subject to oversight from the institutional review board. Patient consent was not required; the project adhered to all applicable regulations and ethical considerations, ensuring the protection of patient confidentiality and privacy. HIPAA-compliant methods were used for data management. The Standards for Quality Improvement Reporting Excellence 2.0 guidelines for reporting practice improvement work were followed [10].

### Model for improvement

The RITE (Realizing Improvement Through Team Empowerment) methodology, renamed ImPower by the American College of Radiology (ACR), served as the process improvement framework. This approach promotes collaboration within multidisciplinary teams, equipped with data resources to tackle specific performance challenges. Progress is monitored through weekly meetings and bi-weekly reporting sessions. The program follows the Plan-Do-Study-Act (PDSA) cycle, an iterative, structured approach for testing and refining changes [11].

### Team

The multidisciplinary team formed for this project includes a diverse group of experts, such as board-certified pediatric body radiologists, MRI physicist, MR technologists, a Clinical Quality & Operations Improvement Specialist, an Human Factors Engineer, data scientists, and research personnel. This collaboration across disciplines enabled the integration of clinical expertise, technical innovation, and data-driven analysis to drive meaningful improvements in patient care.

### Process mapping

**Fig. 2:**
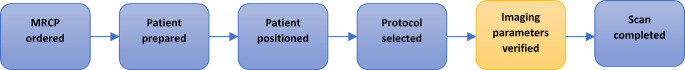
Workflow process for MRCP exam from order to completion

The team conducted Gemba walks, defined as systematic onsite observations, in the MRI units where MRCP exams are acquired. During these visits, they carefully observed and documented each step of the process, from the initial placement of the MRCP order to the final completion of the scan (Fig. 2). This hands-on approach allowed the team to interview radiologists and technologists and investigate utilized scanners to gain valuable insights into the workflow and identify areas for potential improvement.

### Root cause analysis

**Fig. 3:**
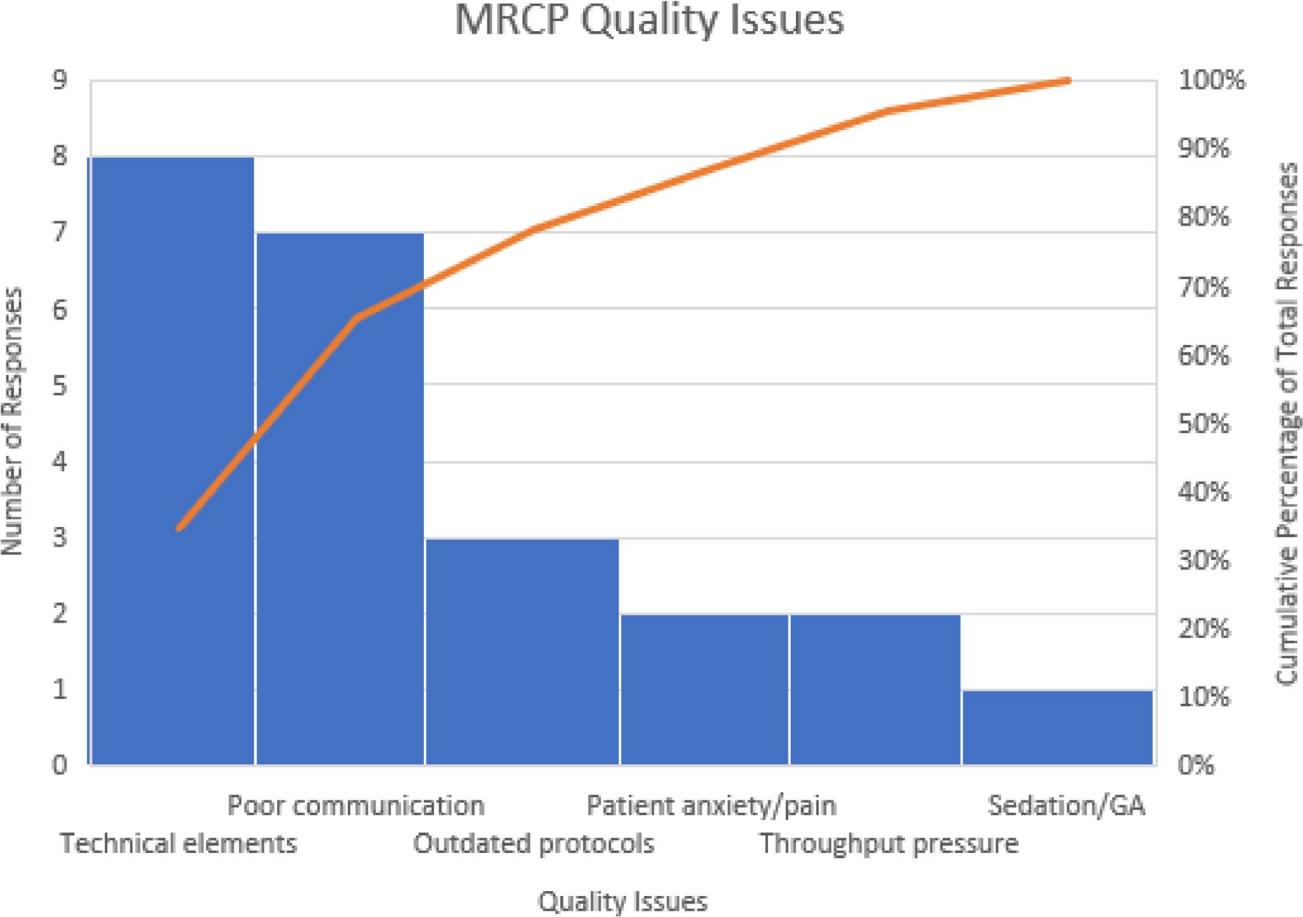
Factors contributing to sub-optimal MRCP imager quality. The trend line represents the cumulative percentage of various factors affecting MRCP image quality, with technical elements, poor communication, and outdated protocols being the most significant contributors

Root cause analysis identified that the quality of MRCP images was compromised due to protocols varied across multiple scanners resulting in the inconsistent application of technical parameters [12]. The “respiratory triggering” technique was not consistently included in 3D T2-weighted sequence in the MRCP protocol, resulting in suboptimal images. Additionally, the “liver dome” or “phase scout” navigator techniques were also applied variably, with some technologists omitting them altogether. As a result, the MRCP protocols required updates to standardize practices within the department. Moreover, inconsistent communication and feedback between radiologists, trainees, and technologists further contributed to the acceptance of low-quality MRCP images (Fig. 3).

### Key drivers

**Fig. 4:**
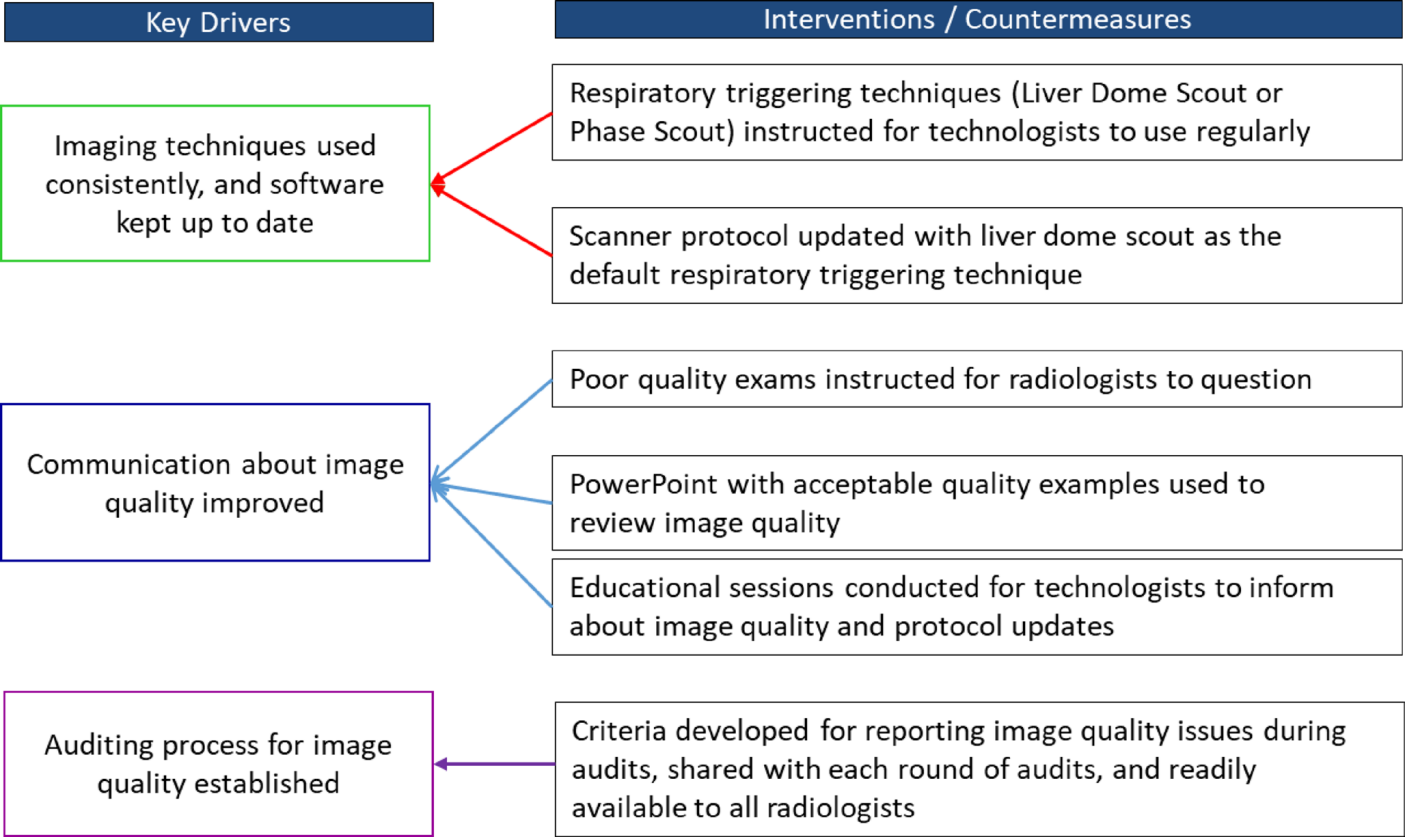
Key drivers and interventions to improve image quality of MRCP exams

The key drivers for improving MRCP image quality focus on optimizing the entire imaging process. This includes enhancing communication between radiologists, technologists, and trainees, as well as ensuring consistent use of imaging techniques and keeping protocols up to date. Furthermore, establishing a regular auditing process for image quality has proven essential in maintaining consistent standards and promoting continuous improvement (Fig. 4).

### Interventions (PDSA)

#### MRCP protocol updates and technologist education

**Fig. 5:**
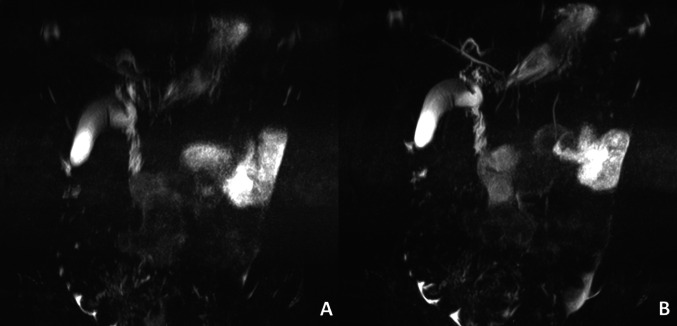
3D T2-weighted MRI sequences of the same patient. **A** represents the 3D T2-weighted MRI sequence from an MRCP exam without the use of the respiratory trigger technique. **B** represents the 3D T2-weighted MRI sequence from an MRCP exam with the use of the respiratory trigger technique. Notice the detailed view of biliary and pancreatic ducts on **B** in comparison to blurry views on **A**

In May 2023, the team updated MRCP protocols across all scanners to enhance imaging technique consistency. Technologists were instructed to incorporate respiratory triggering techniques, using either a liver dome scout or phase scout, to minimize motion artifacts from breathing (Fig. 5). By May 2024, the liver dome technique was set as the default setting on scanners, ensuring standardized practices.

To support these changes, the MRI physicist on the team conducted educational sessions for technologists, offering both one-on-one training and didactic presentations during technologists’ recurring meetings. These sessions covered the updated protocols, including how to implement respiratory triggering and operate the scanners using the updated techniques.

### Improving communication on image quality

In May 2023, the team focused on improving communication between radiologists, trainees, and technologists. Radiologists and trainees were encouraged to be more proactive in questioning scans of poor quality, rather than passively accepting them. This created a feedback loop, enabling technologists to quickly address and correct issues identified by radiologists and trainees, fostering a more collaborative approach toward achieving consistent, high-quality imaging.

### MRCP exam quality auditing

Starting in March 2024, the team established clear criteria for auditing MRCP image quality to ensure consistent scoring, replacing the previous approach that relied on undefined measures and subjective scoring based on the radiologists’ experience. Criteria for reporting image quality issues during audits were developed based on the work of He et al. [13]. These criteria involved scoring image diagnostic confidence on a 5-point Likert scale, where 1 represented the absence of diagnostic confidence and 5 indicated excellent quality or full diagnostic confidence.

The assessment focused on three major findings:

1. Artifacts: Evaluating the presence, frequency, and severity of artifacts, from severe motion and wrap artifacts to a complete absence of artifacts.
2. Background Suppression on 3D SPACE sequence: Assessing the extent of background suppression, ranging from very strong background signal that precluded image interpretation to excellent suppression with no impact on diagnostic quality.
3. Visibility of Key Ducts: Reviewing the visibility of the common bile duct, common hepatic duct, hepatic duct branches, and the pancreatic duct.

### Study of the interventions

**Fig. 6:**
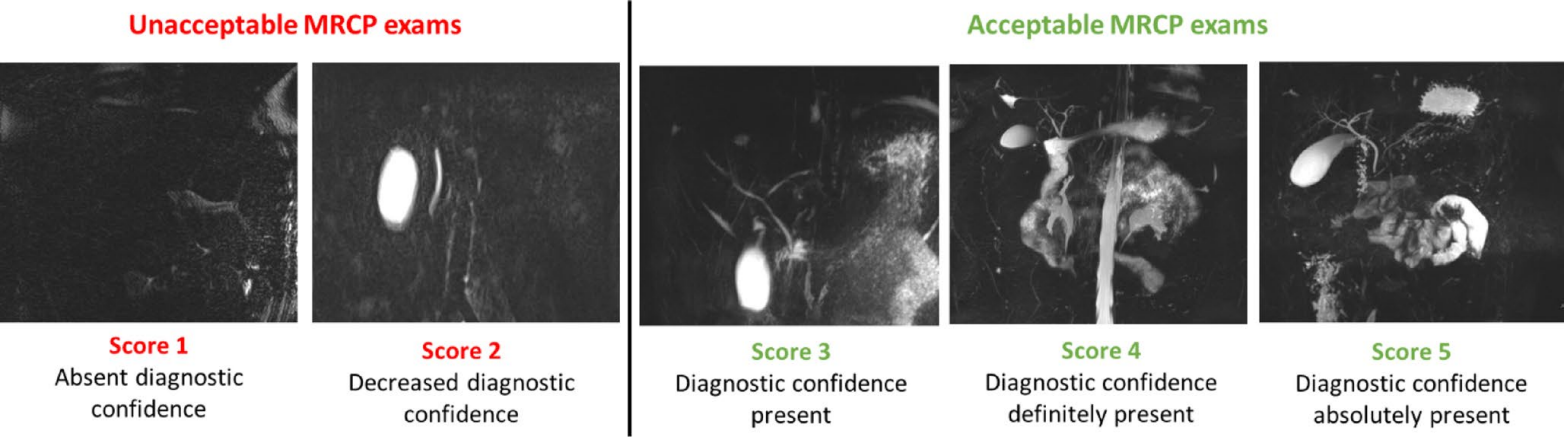
MRCP exams with different quality scores from score 1 (absence of diagnostic confidence) to score 5 (diagnostic confidence absolutely present)

The analytics dashboard, the visual interface that displays key data metrics, was updated to store MRCP exams information including details about the protocol followed and respiratory triggering. The primary outcome measure of the project was the percentage of MRCP exams with an acceptable image quality. To ensure consistency in image quality assessment, multiple pediatric body radiologists, with experience ranging from 1 to 30 years, reviewed each MRCP exam using the 5-point Likert scale detailed above. The average score from the reviewers was calculated to determine the overall image quality score for each exam, ensuring a more balanced and reliable evaluation. Scores were grouped into two categories: 1–2 as “sub-optimal quality” and 3 or higher as “acceptable quality” (Fig. 6).

Additionally, the primary process measure of the project focused on the percentage of MRCP exams that utilized the “Liver Dome Scout” for respiratory triggering.

The primary outcome and process measures were evaluated using control P-charts to assess the impact of interventions over time. These charts display proportions of noncontinuous data with centerlines representing the mean for each distinct project phase, allowing differentiation between common-cause and special-cause variation. Outcome measures were assessed only during weeks when interventions were applied consistently. Special-cause variation was identified using standard control chart rules, including: any single point outside the control limits; four out of five successive points more than 1 standard deviations (SD) from the mean on the same side; two out of three consecutive points more than 2 SD from the mean on the same side of the centerline; eight or more consecutive points on one side of the centerline (a true process shift); or six or more points in a consistently increasing or decreasing trend [14].

## Results

The project launched in May 2023 and spanned 17 months through September 2024. Over the course of the project, a total of 308 MRCP exams were reviewed.

**Fig. 7:**
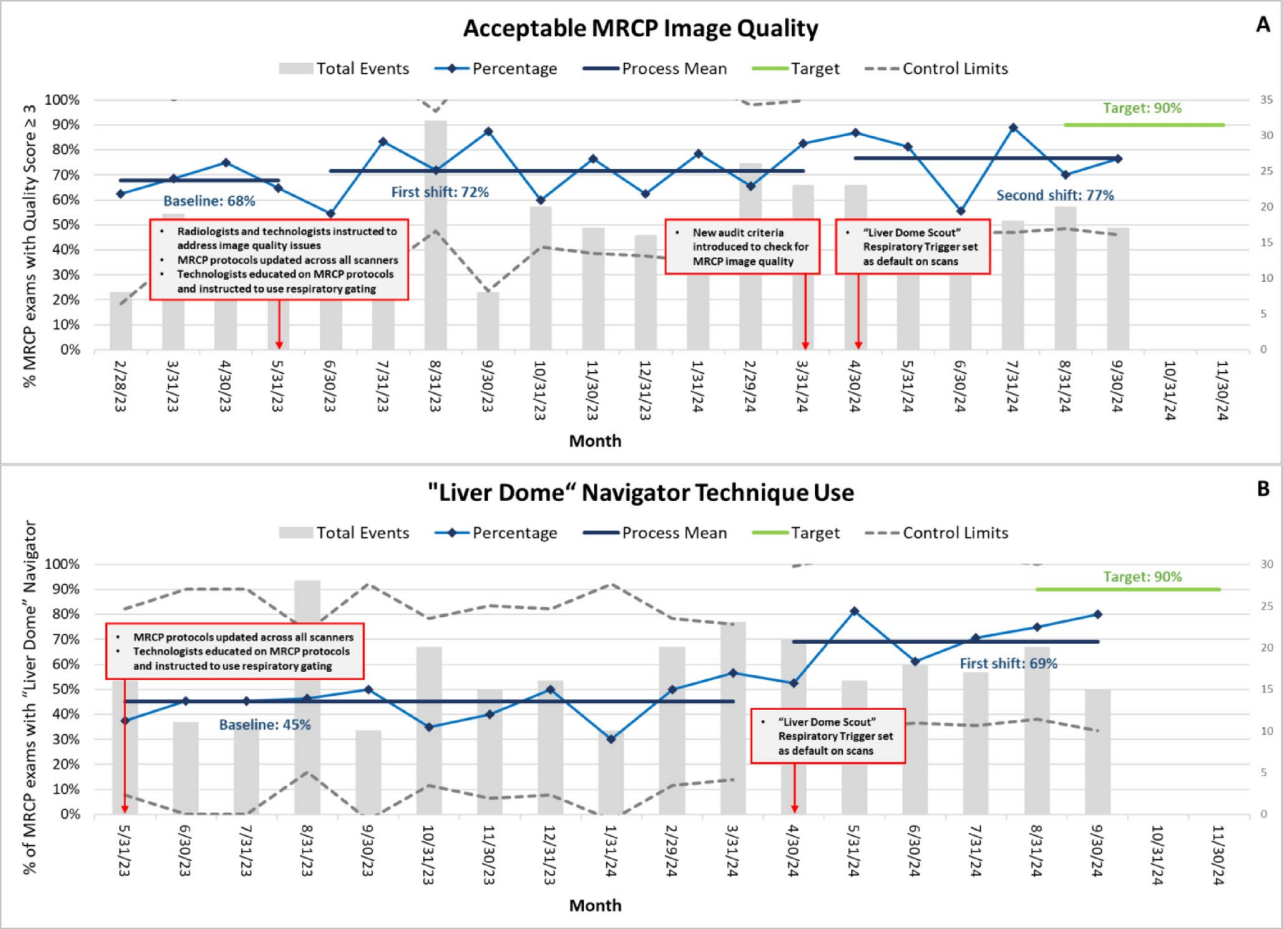
“**A**” represents the *Primary outcome measure*: Variation in the percentage of MRCP exams with an acceptable image quality (≥ 3 score) following the implementation of interventions. “**B**” represents the *Primary process measure*: Variation in the percentage of MRCP exams utilizing the respiratory trigger technique with liver dome scout following the implementation of interventions

Between February and April 2023, prior to the initiation of this QI effort, 68% of MRCP exams were rated as having acceptable image quality. After introducing initial interventions in May 2023 and conducting PDSA cycles to refine and implement further interventions, the percentage of MRCP with acceptable image quality exams increased to 77% by September 2024. Post-intervention data showed four out of five points above + 1 SD (73.13%) and two out of three points above + 2 SD (78.6%). However, there were neither eight consecutive points on one side of the centerline nor six points showing a consistent trend (Fig. 7A).

Starting in May 2023, all MRI scanners were updated with standardized MRCP protocols, and technologists were instructed to use respiratory triggering. From May 2023 to March 2024, the baseline percentage of MRCP exams using the liver dome scout for respiratory triggering was 45%. After the liver dome scout was set as the default trigger on the protocols saved on scanners in April 2024, this percentage increased to 69% by September 2024. Post-intervention data points showed that four out of five successive values exceeded + 1 SD (52%), and two out of three successive values exceeded + 2 SD (59.8%). However, there were no sequences of eight consecutive points above the mean, nor were there six successive points showing a consistent trend (Fig. 7B).

## Discussion

This project improved MRCP exams with an acceptable image quality from 68 to 77% after multiple PDSA cycles. Although the observed increase indicates special cause variation (four out of five successive points more than 1 SD from the mean on the same side and two out of three consecutive points more than 2 SD from the mean on the same side of the centerline), it did not meet the statistical process control criteria required to confirm a true process shift (8 successive data points above the mean). Despite not meeting the set goal of increasing the percentage of acceptable quality MRCP exams to 90%, this initiative successfully enhanced focus on protocol standardization, respiratory triggering instructions, and communication between team members, including radiologists, technologists, and other health care professionals. This underscores the importance of team-based problem-solving in QI efforts, in line with the principles of the RITE methodology [15].

**Fig. 8:**
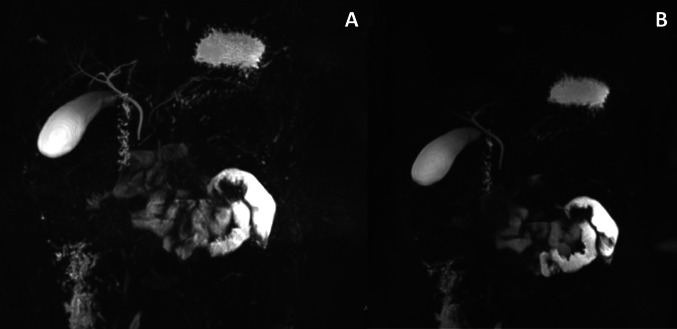
T2-weighted 3D SPACE MRCP exam of a 9 year old boy. “**A**” represents T2 3D SPACE MRCP acquisition using the respiratory triggering technique. “**B**” represents T2 3D SPACE MRCP image acquisition using the Breath-Hold technique

The use of the respiratory triggering technique for MRCP acquisition, particularly in younger children, significantly enhances image quality by improving both signal-to-noise and contrast-to-noise ratios [15, 16]. Although some literature suggests that the breath-hold technique may yield higher-quality 3D SPACE MRCP images [17], our experience indicates otherwise. As demonstrated in the example below, respiratory triggering provides higher image quality, with improved visualization of the pancreatic vessels and biliary tree (Fig. 8). In contrast to the respiratory-triggering technique, breath-hold acquisition utilizes a single-shot fast spin echo technique allowing for rapid image capture during a single breath-hold hence limiting the time available for data acquisition. This time constraint inherent to the breath hold technique can negatively impact spatial resolution, meaning the ability to see fine details in the image. As a result, breath-hold sequences typically offer lower spatial resolution compared to respiratory-triggered acquisitions [18].

Several challenges emerged during the project that prevented the achievement of the targeted goal of 90% within the designated timeframe. A key barrier was the lack of standardized protocols across multiple MRI scanners, particularly due to inconsistencies in applying technical parameters. These inconsistencies led to suboptimal image quality, even after the initial interventions were implemented. Addressing them required both technical and human-centered solutions. On the technical side, we tested setting optimized techniques as default options on scanners during the later stages of the project. In parallel, we introduced human factors interventions, including ongoing, comprehensive technologist education on the effective use of these techniques. However, due to workload demands and varying schedules, educational efforts were inconsistent, and some technologists may not have been fully aware of how to apply the correct techniques optimally. Despite these efforts, inconsistencies persisted. Nonetheless, the education initiatives helped build skills and increase awareness of the importance of protocol adherence.

Effective communication between radiologists, trainees, and technologists played a crucial role in identifying factors influencing image quality, establishing a feedback loop aimed at sustainable improvement. Radiologist feedback empowered technologists to take a more proactive role in ensuring high-quality imaging, reinforcing a culture of continuous improvement. However, maintaining ongoing communication between radiologists and technologists proved burdensome for both groups. While essential for enhancing image quality, frequent interactions risked disrupting workflow and diverting radiologists’ attention from interpreting exams. In some cases, this may have contributed to radiologists accepting suboptimal image quality due to high workloads and limited time to delay interpretation [19].

The use of regular audits and standardized scoring played a pivotal role in ensuring consistency and providing a structured framework for evaluating MRCP image quality. The systematic approach allowed for real-time adjustments, and the regularity of the audits ensured prompt resolution of any issues. The standardized scoring provided clear, objective benchmarks for both current performance and future goals. Establishing such auditing processes is vital for sustaining long-term improvements in MRCP image quality, as it fosters a culture of accountability and provides ongoing data to inform decision-making [19].

## Limitations

This project did not measure the difference in image acquisition time before and after implementing the interventions. Assessing whether these interventions prolonged scan times would have provided valuable insights. In addition, factors influencing motion, including age of the patient and anesthesia use during MRCP exams were not recorded. Future efforts by the team will include an analysis of these variables to ensure that workflow efficiency is maintained while enhancing image quality.

## Application in resource-limited settings

Institutions with limited resources can adapt these interventions through cost-effective approaches. Protocols can be standardized using existing scanner capabilities, such as respiratory gating, even in the absence of advanced navigator tools. Training can be delivered through low-cost methods like virtual or group-based sessions, supported by recorded tutorials to ensure sustainability. If digital dashboards are unavailable, manual auditing using paper forms or spreadsheets can still effectively track image quality. Communication between radiologists and technologists can be enhanced through simple strategies such as regular image review huddles, without the need for advanced infrastructure. Lastly, a phased implementation of protocol updates, beginning with a single scanner, allows for gradual adoption and minimizes the burden on limited resources.

## Conclusion

This quality improvement project successfully enhanced the image quality of 3D MRCP exams at a tertiary children’s hospital. Key interventions, including protocol updates, technologist education, and improved communication between radiologists and technologists, were integral to achieving these improvements. The project’s outcomes underscore the importance of multidisciplinary collaboration, continuous education, and regular auditing in sustaining high quality imaging.

## Data Availability

No datasets were generated or analysed during the current study.
